# Prevalence and prognosis of non-ischemic patterns of late gadolinium enhancement in older adults by cardiovascular MR in the ICELAND-MI study

**DOI:** 10.1186/1532-429X-18-S1-O61

**Published:** 2016-01-27

**Authors:** Sujata M Shanbhag, Thor Aspelund, Anders M Greve, Gudmundur Thorgeirsson, Erik B Schelbert, Jie J Cao, Sigurdur Sigurdsson, Peter Kellman, Gudny Eiriksdottir, Tamara Harris, Lenore Launer, Vilmundur Gudnason, Andrew E Arai

**Affiliations:** 1 Icelandic Heart Association, Kopavogur, Iceland; 2 Public Health Sciences, University of Iceland, Reykjavik, Iceland; 3 Gentofte Hospital, Copenhagen, Iceland; 4 University of Iceland, Reykjavik, Iceland; 5 Division of Cardiology, UPMC Heart and Vascular Institute, Pittsburgh, PA USA; 6 Cardiology, St. Francis Hospital, Roslyn, NY USA; 7 NIH/NHLBI, Bethesda, MD USA; 8 National Institute on Aging, NIH, Bethesda, MD USA

## Background

Late gadolinium enhancement (LGE) can detect and discriminate myocardial scar/fibrosis of ischemic and non-ischemic etiologies. Our objective was to determine the prevalence and prognosis for ischemic and non-ischemic patterns of LGE in a community-based sample of older adults.

## Methods

ICELAND-MI is a nested cohort of the Age, Gene/Environment Susceptibility-Reykjavik Study of community-dwelling older adults that intentionally over-sampled diabetic subjects. After excluding subjects with pre-existing heart failure, the cohort size was 900. CMR was used to detect myocardial infarction (MI), major patterns of non-ischemic patterns of LGE as defined by Vöhringer (Herz 2007;32:129-37), and minor patterns of non-ischemic LGE including LGE near the aortic root, mitral annulus, or right ventricular insertion points. The composite end-point was adjudicated hospitalization for heart failure and death.

## Results

**Figure 1:**
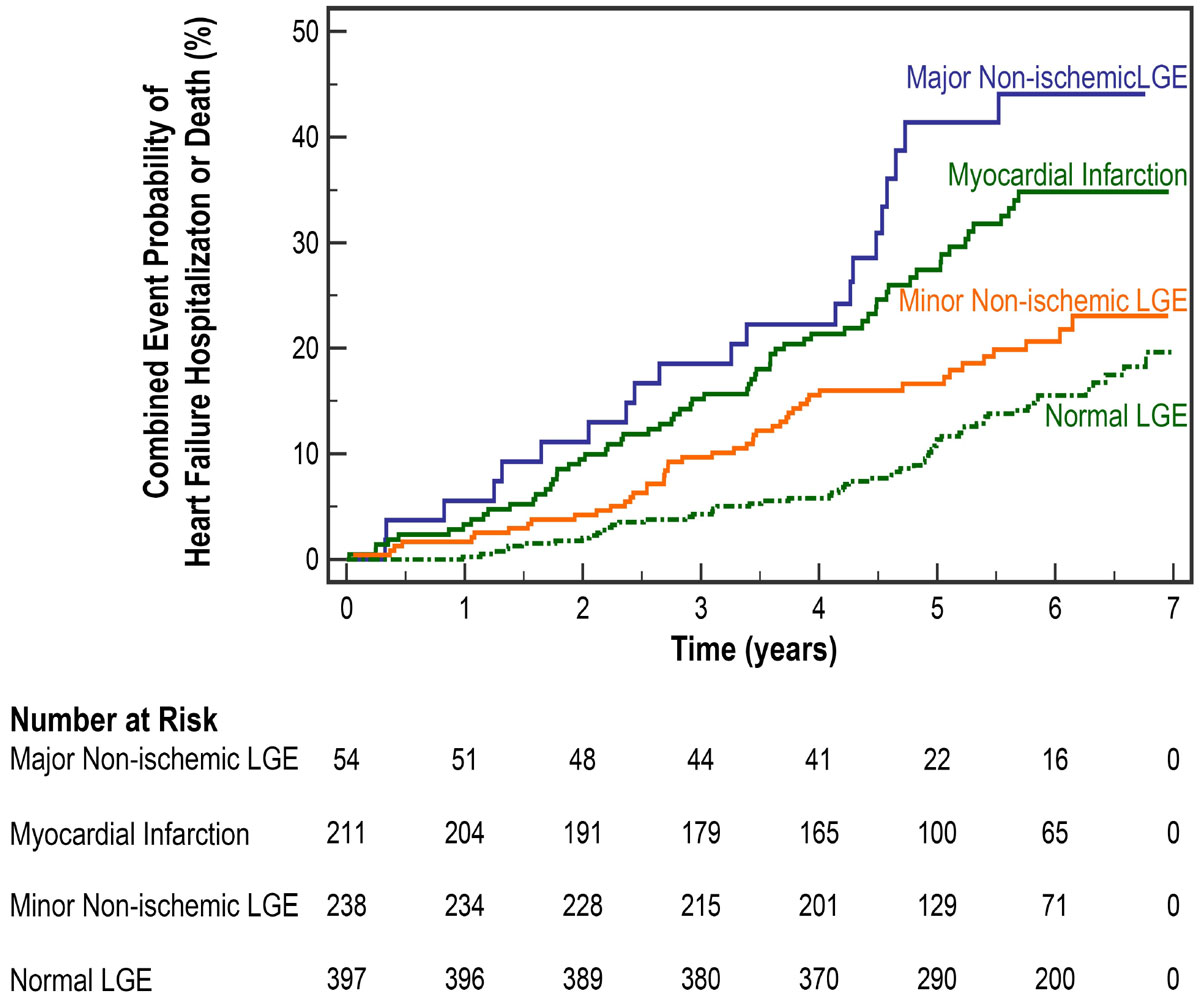
**Kaplan-Meier Event Rates for Subjects with Major Non-ischemic LGE, MI, Minor Non-ischemic LGE versus Normal LGE**.

The median age was 76 (IQR 72-81), 48% were male, and 35% had diabetes. The prevalence of MI, major non-ischemic patterns of LGE, and minor non-ischemic patterns of LGE were 23.4%(N = 211), 6.0%(N = 54), and 26.4%(N = 238) respectively. Major non-ischemic LGE demonstrated the highest risk (HR 3.5, p < 0.0001, Figure 1), MI had similar risk (HR 2.5, p < 0.0001), and minor non-ischemic LGE had lower but significantly higher risk (HR1.5, p = 0.03) compared to those without LGE. Controlling for age, gender, LVEF, diabetes, and hypertension, major non-ischemic LGE remained strongly predictive of the composite endpoint (HR 2.3, p = 0.001) while minor non-ischemic patterns of LGE were of borderline significance (p = 0.07).

## Conclusions

Subjects with major non-ischemic LGE patterns are at increased risk of developing heart failure and death.

